# Expression of VEGF and Flk-1 and Flt-1 Receptors during Blood-Brain Barrier (BBB) Impairment Following *Phoneutria nigriventer* Spider Venom Exposure

**DOI:** 10.3390/toxins5122572

**Published:** 2013-12-18

**Authors:** Monique C. P. Mendonça, Edilene S. Soares, Leila M. Stávale, Catarina Rapôso, Andressa Coope, Evanguedes Kalapothakis, Maria Alice da Cruz-Höfling

**Affiliations:** 1 Department of Pharmacology, Faculty of Medical Sciences, State University of Campinas (Unicamp) Campinas, SP 13083-887, Brazil; E-Mail: monique.cpm@gmail.com; 2 Department of Histology and Embryology, Institute of Biology, State University of Campinas (Unicamp) Campinas, SP 13083-863, Brazil; E-Mails: esiqueirasoares@gmail.com (E.S.S.); leilinhabio@yahoo.com.br (L.M.S.); cataraposa@gmail.com (C.R.); 3 Cell Signaling Laboratory, Faculty of Medical Sciences, State University of Campinas (Unicamp), Campinas, SP 13081-970, Brazil; E-Mail: andressacoope@gmail.com; 4 Department of General Biology, Institute of Biological Sciences, Federal University of Minas Gerais (UFMG), Belo Horizonte, MG 31270-901, Brazil; E-Mail: kalapothakis@gmail.com

**Keywords:** hippocampus, junctional proteins, Neu-N, VEGF, VEGF receptors

## Abstract

Apart from its angiogenic and vascular permeation activity, the vascular endothelial growth factor (VEGF) has been also reported as a potent neuronal protector. Newborn rats with low VEGF levels develop neuron degeneration, while high levels induce protective mechanisms in several neuropathological conditions. *Phoneutria nigriventer* spider venom (PNV) disrupts the blood-brain barrier (BBB) and causes neuroinflammation in central neurons along with excitotoxic signals in rats and humans. All these changes are transient. Herein, we examined the expression of VEGF and its receptors, Flt-1 and Flk-1 in the hippocampal neurons following envenomation by PNV. Adult and neonatal rats were evaluated at time limits of 2, 5 and 24 h. Additionally, BBB integrity was assessed by measuring the expression of occludin, β-catenin and laminin and neuron viability was evaluated by NeuN expression. VEGF, Flt-1 and Flk-1 levels increased in PNV-administered rats, concurrently with respective mRNAs. Flt-1 and Flk-1 immunolabeling was nuclear in neurons of hippocampal regions, instead of the VEGF membrane-bound typical location. These changes occurred simultaneously with the transient decreases in BBB-associated proteins and NeuN positivity. Adult rats showed more prominent expressional increases of the VEGF/Flt-1/Flk-1 system and earlier recovery of BBB-related proteins than neonates. We conclude that the reactive expressional changes seen here suggest that VEGF and receptors could have a role in the excitotoxic mechanism of PNV and that such role would be less efficient in neonate rats.

## 1. Introduction

Spider venoms are rich sources of low molecular mass compounds with a wide range of pharmacological effects on ion channels, neurotransmitter receptors and transporters and consequently on synaptic transmission [1]. *Phoneutria nigriventer* spider venom (PNV) contains a plethora of pharmacologically active peptides which interfere with the physiology of the tetrodotoxin (TTX)-sensitive Na^+^ channels and block the Ca^2+^ and K^+^ channels. Therefore PNV-induced changes in the concentration of these ions affect the glutamate transporter, resulting in increased extracellular glutamate concentration [2,3]. Human victims of *P. nigriventer* spider bites and experimental animals display neuroexcitatory symptoms, which may include convulsion in severe cases, generally in children [4]. Studies have shown that PNV presence in the blood flow induces blood-brain barrier (BBB) breakdown in the hippocampus of rats [5] by enhanced transendothelial microtubule-mediated vesicular transport [6], or by displacement and/or decrease of proteins that control the paracellular pathway [7]. These effects are accompanied by transient neurotoxic manifestations. Despite clinical reports and experimental studies showing that *P. nigriventer* spiderenvenoming causes neurotoxic manifestations and induces permeation of the BBB, little is known about molecular mechanisms triggered in the brain shortly after envenomation.

Increased BBB permeability has been associated with increased expression of angiogenic factors, the most notable of which is the vascular endothelial growth factor (VEGF) [8]. VEGF action results from binding to VEGFR1 and VEGFR2, also known as the Fms-like tyrosine kinase 1 (Flt-1) and fetal liver kinase 1/kinase insert domain receptor (Flk-1/KDR), respectively [9]. Increased VEGF levels have been described in brain repair [10] and in many pathological events affecting the central nervous system [11]. BBB disruption has been associated with pathologic angiogenesis in patients suffering from intractable temporal lobe epilepsy accompanied by overexpression of VEGF in neurons and Flk-1 in endothelial cells [12]. It was thus considered of interest to investigate whether or not *P. nigriventer* experimental envenomation is accompanied by expressional changes in the VEGF/Flt-1/Flk-1 system.

Since the hippocampus is rich in glutamate receptors and transporters and, as part of the temporal lobe is involved in the etiopathogenesis of convulsion-like events like those induced by PNV [4], it is important to focus on this region as a PNV target and seek possible ongoing molecular mechanisms coursing with the BBB permeation [13]. Thus far, studies on the BBB disruption by PNV have used adult rats, but age-related differences have been reported for humans [4] and recently reported for rats [14]. Herein, a possible age-dependent differential response in relation to VEGF involvement is investigated using neonate and adult rats. 

This study will shed light on VEGF, Flt-1 and Flk-1 response generated by circulating *P. nigriventer* spider venom, running in parallel with the BBB breakdown. The fact that the effects are short-lived present good prospects for future studies aiming to promote transient BBB opening for therapeutic purposes.

## 2. Results

Irrespective of age, animals that were administered saline were normal in their feeding and behavioral habits in cages, whereas animals administered PNV displayed unevenly the toxic manifestations described by other authors, including piloerection, shivering, hypersalivation, respiratory distress, spastic hindlimb paralysis and tonic convulsion [5,13].

### 2.1. Blood-Brain Barrier-Associated Proteins: Occludin, *β*-Catenin, Laminin

**Figure 1:**
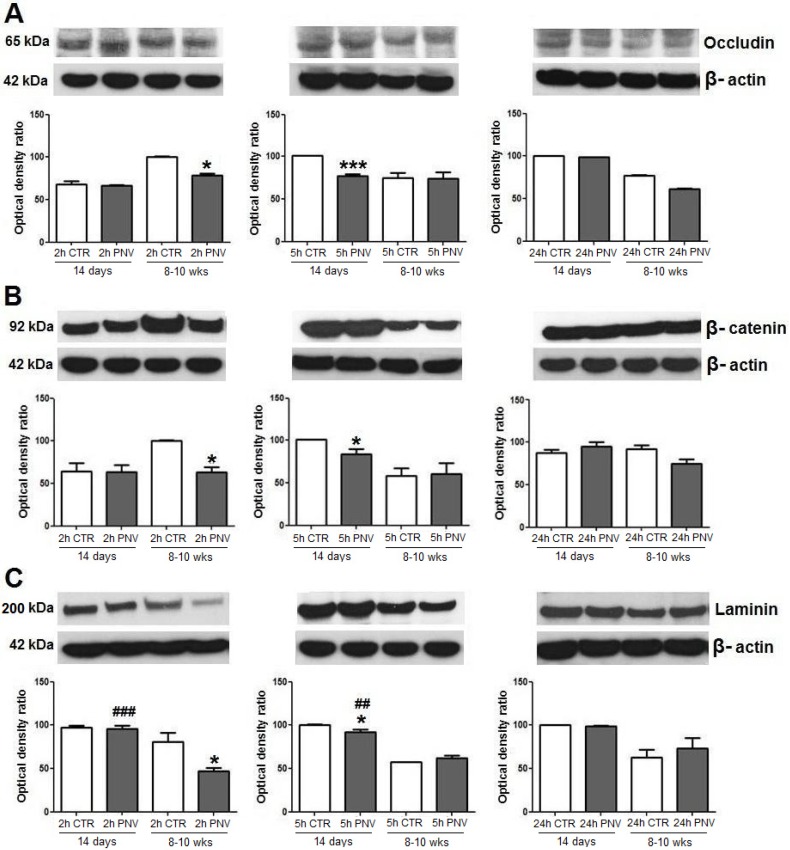
Immunoblots of occludin (**A**), β-catenin (**B**) and laminin (**C**). *Phoneutria nigriventer* (PNV) intra-peritoneal (i.p) injection induced significant decreases of all three proteins at 2 h for adults, whereas at 5 h for neonates. * *p* ≤ 0.05 and *** *p* ≤ 0.001 denote significant decreases relative to controls; ^##^ *p* ≤ 0.01 and ^###^ *p* ≤ 0.001 indicate PNV-treated neonates with higher increase in laminin expression than their adult counterpart at 2 and 5 h. Student *t*-test; data are shown as means ± SEM.

Occludin, β-catenin and laminin showed a relatively similar pattern of response to PNV envenomation. All decreased significantly and transiently in adult and neonate rats exposed to PNV, with such decreases occurring earlier in adults than in neonates, after which they exhibited recovery towards baseline (Figure 1A–C).

Relative to respective control levels, occludin expression decreased significantly earlier in adults (2 h) than in neonates (5 h) and showed a 28% decrease (* *p* ≤ 0.05) compared with a 30% decrease in neonates (*** *p* ≤ 0.001); there was no difference between PNV *vs*. controls in protein expression at 24 h (Figure 1A). PNV-administered adult rats showed immediate (2 h) 55% downregulation of β-catenin (* *p* ≤ 0.05), whereas downregulation in neonates occurred at 5 h and was 20% in magnitude (* *p* ≤ 0.05). These changes were transitory given that at 24 h protein expression was close to baseline for rats of both ages (Figure 1B). The reduction of laminin expression was higher (73%, * *p* ≤ 0.05) and earlier (2 h) in adult rats administered PNV than in matched neonates (5 h, 9% reduction, * *p* ≤ 0.05) (Figure 1C). Laminin expression was lower in adult rats in comparison with neonates for both control and PNV groups. There were age-related differences in laminin expression between PNV-treated rats at 2 h (^###^ *p* ≤ 0.001) and 5 h (^##^ *p* ≤ 0.01), as illustrated in Figure 1.

### 2.2. Immunohistochemistry: PNV Increased VEGF, Flt-1, Flk-1 Immunostaining

**Figure 2:**
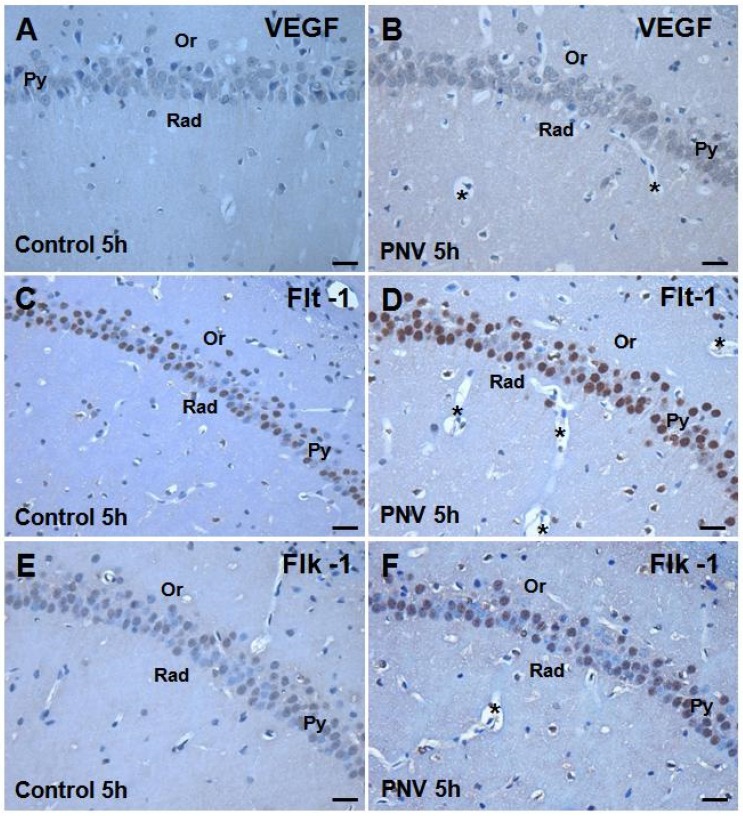
CA1 subfield of 8–10-week-old rats: composite photomicrographs of hippocampal coronal sections stained for localization of anti-VEGF, anti-Flt-1 and anti-Flk-1 and using the immunoperoxidase technique. (**A**,**C**,**E**) are sections from controls (saline-treated) and (**B**,**D**,**F**) are from PNV-treated adult rats 5 h after i.p. injection. PNV increased the immunoreactivity of VEGF, Flt-1 and Flk-1. Py = stratum pyramidale; Or = stratum oriens; Rad = stratum radiatum; * = microvessels with perivascular edema. Scale Bars = 25 µm.

Figure 2 illustrates the VEGF, Flt-1 and Flk-1 staining pattern in the CA1 subfield of the hippocampus of adult rats taken at 5 h after i.p. administration of saline or PNV. Under control conditions (saline), VEGF was expressed in pyramidal neuron bodies and dendrites of the CA1, CA2 and CA3 and granule neurons of the dentate gyrus (DG). Flt-1 and Flk-1 receptors were mainly expressed in the nuclei of pyramidal and granule neurons. Flt-1 staining was also seen in neuron processes. Flt-1 staining was stronger than that of Flk-1. Such a pattern was mirrored by CA2, CA3 and DG hippocampal subfields for rats of both ages.

The immunostaining of VEGF and Flt-1 and Flk-1 receptors was increased in all the subfields of hippocampus of PNV-treated rats of both ages. Figure 2 (panels A to F) depicts CA1 subfield of adult rats injected with saline and PNV as representative of the staining pattern exhibited by the other subfields. Consistently, anti-Flt-1 staining increased more prominently than VEGF and Flk-1. While several nuclei were Flk-1 negative (Figure 2F), practically all nuclei were Flt-1 positive (Figure 2D) and all neuron bodies were VEGF positive (Figure 2B). A similar staining pattern was observed in PVN-treated P14 rats (not shown).

It should be mentioned that the microcirculation vessels of PNV-administered animals presented perivascular edema in all the hippocampal subfields examined (Figure 2B,D,F as representatives) confirming previous quantitative studies [5,13,14].

### 2.3. Western Blotting and Real-Time Polymerase Chain Reaction (PCR): Temporal and Age-Related Differences

VEGF, Flt-1 and Flk-1 expression in the hippocampus homogenate (comprising all ammonic subfields together, CA1, CA2, CA3) and DG showed time- and age-dependent differences in PNV-administered rats.

#### 2.3.1. VEGF, Flt-1 and Flk-1 Immunoblot Quantification

**Figure 3:**
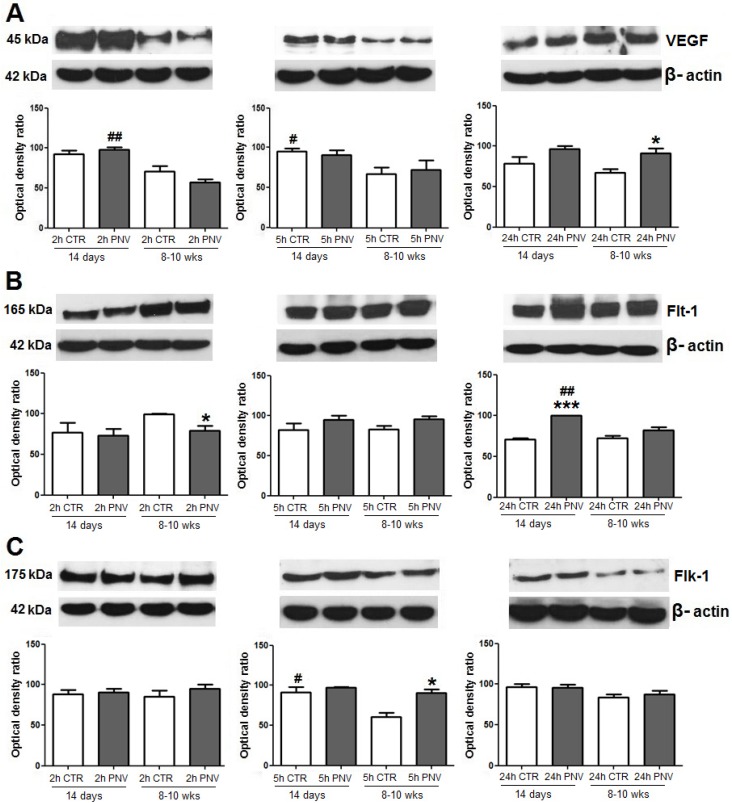
Western blot signals were densitometrically quantified and normalized to an internal standard (β-actin). VEGF (**A**), Flt-1 (**B**) and Flk-1 (**C**) expressions in PNV-treated samples (1.7 mg/kg) relative to control (CTR); * *p* ≤ 0.05 and *** *p* ≤ 0.001 indicate significant difference relative to respective controls; ^#^ *p* ≤ 0.05 and ^##^ *p* ≤ 0.01 denote significant age-related differences between control (CTR) or PNV-treated groups at corresponding time-point. Student *t-*test; data were shown as means ± SEM.

In the hippocampus of PNV-treated adult rats, VEGF increased by 36% at 24 h, but not for neonates (Figure 3A; * *p* ≤ 0.05). VEGF expression, basally and after venom exposure, was higher in neonates than in adults, being 70% significantly higher at 2 h in neonates than in envenomed adults (^##^ *p* ≤ 0.01) and 42% significantly higher at 5 h in neonates than control adults (^#^ *p* ≤ 0.05).

At 24 h, Flt-1 expression was 40% higher in PNV-treated P14 rats (*** *p* ≤ 0.001), whereas it was 25% lower in PNV-treated adults at 2 h relative to baseline (Figure 3B; * *p* ≤ 0.05). At 24 h post-PNV, Flt-1 expression was higher in neonates than in adults (^##^ *p* ≤ 0.01). Flk-1 expression level was upregulated by 50% in adult rats after 5 h, relative to baseline (* *p* ≤ 0.05, Figure 3C), and remained unchanged in neonates.

Three-way ANOVA analysis revealed interaction between treatment *vs*. time variables so influencing the expression of VEGF and Flt-1 (* *p* ≤ 0.05).

#### 2.3.2. VEGF, Flt-1 and Flk-1 mRNAs (qPCR)

**Figure 4:**
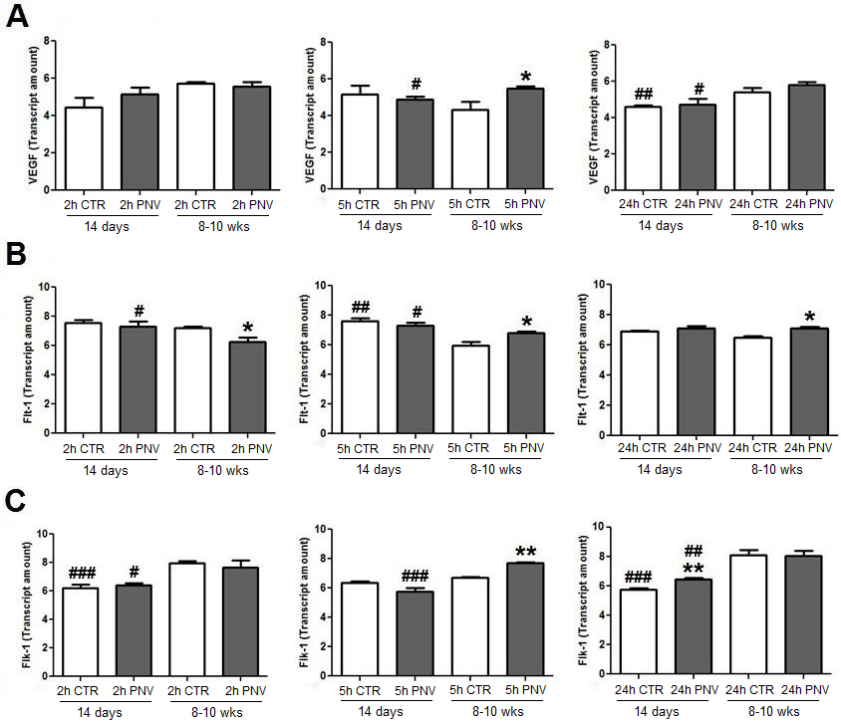
Quantitative real-time polymerase chain reaction (PCR) analysis quantified and normalized to endogen control (GAPDH). VEGF (**A**), Flt-1 (**B**) and Flk-1 (**C**) mRNAs expression at time-points after PNV (1.7 mg/kg) or 0.9% saline peritoneal injection. * *p* ≤ 0.05 and ** *p* ≤ 0.01 indicate significant difference relative to respective controls; ^#^ *p* ≤ 0.05, ^##^ *p* ≤ 0.01 and ^###^ *p* ≤ 0.001, denote significant age-related differences between control (CTR) or PNV-treated groups at corresponding time-point. Student *t-*test; data were shown as means ± SEM.

In adult rats treated with PNV, VEGF mRNA showed a 27% increase at 5 h (Figure 4A), Flt-1 mRNA expression showed a 15% decrease at 2 h, followed by increases of 15% and 5% at 5 h and 24 h, respectively (* *p* ≤ 0.05) and Flk-1 mRNA showed a 15% increase at 5 h (** *p* ≤ 0.01, Figure 4B,C). In envenomed neonates, only Flk-1 mRNA expression was 12% above control at 24 h (** *p* ≤ 0.01, Figure 4B,C).

Age-related comparison showed that at 5 and 24 h VEGF mRNA expression in adult rats was, respectively, 12% and 18% above the level found in envenomed neonates (^#^ *p* ≤ 0.05). In contrast, Flt-1 mRNA was higher in neonates than in adults at 2 and 5 h (^#^ *p* ≤ 0.05) and the Flk-1 mRNA level was higher in adults than in neonates at 2, 5 and 24 h (^#^ *p* ≤ 0.05, ^###^ *p* ≤ 0.001 and ^##^ *p* ≤ 0.01, respectively).

The three-way ANOVA analysis showed that interaction between two variables influenced the third variable for expression of VEGF, Flt-1 and Flk-1 mRNAs (* *p* ≤ 0.05).

### 2.4. NeuN Immunohistochemistry and Western Blotting

**Figure 5:**
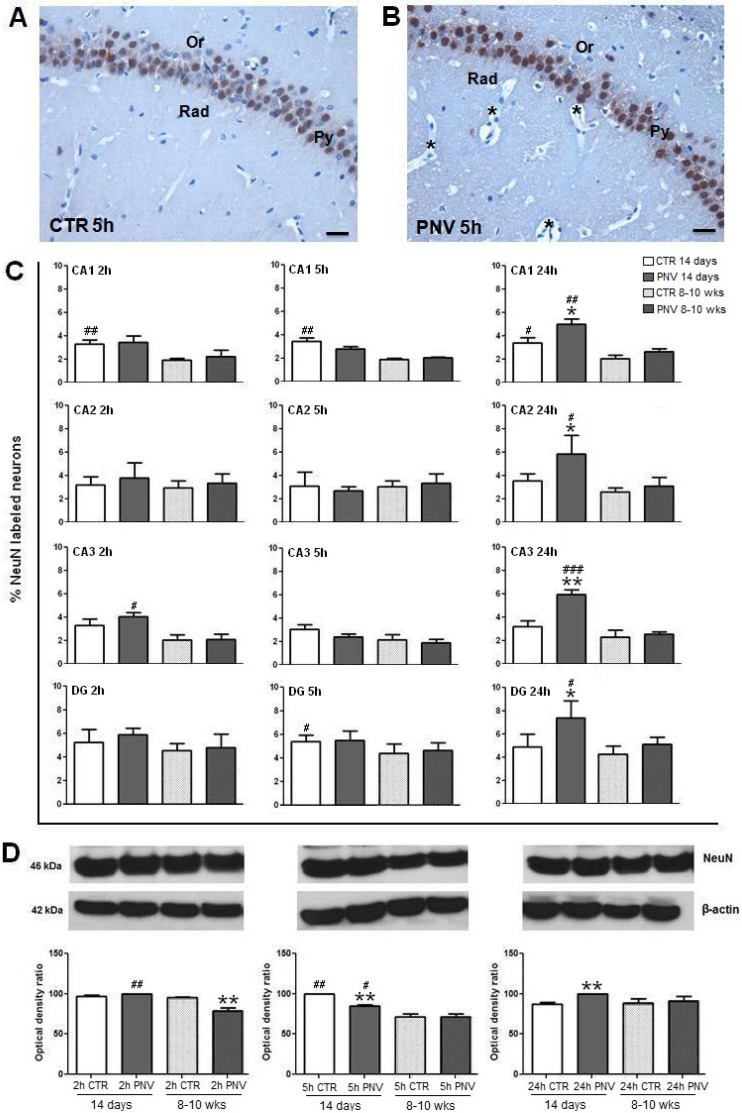
NeuN labeling in the CA2 subfield of 8–10-week-old rats 5 hafter i.p. injection of saline solution (**A**) or PNV (**B**). Py = stratum pyramidale; Or = stratum oriens; Rad = stratum radiatum; * = microvessels with perivascular edema. (**C**) Percentage of pixel density of NeuN-labeled neurons in different time points. (**D**) Immunoblots of NeuN in the hippocampus of i.p.-injected saline or PNV rats at 2, 5 and 24 h. * *p* ≤ 0.05 and ** *p* ≤ 0.01 denote significant decrease of the nuclear marker relative to respective controls; ^#^ *p* ≤ 0.05, ^##^ *p* ≤ 0.01 and ^###^ *p* ≤ 0.001) denote significant difference in NeuN expression between control (CTR) or PNV-treated groups at the corresponding time-point. Student *t*-test; data were shown as means ± SEM. Scale Bars = 25 µm.

NeuN was expressed in the nuclei of pyramidal and granule neurons. Figure 5 illustrates anti-NeuN labeling in the hippocampal CA2 region of an adult rat treated with saline (panel A) and PNV (panel B). Figure 5C shows the time-course quantification of the density of pixels, expressed as percentage, of NeuN-labeled neurons in CA1, CA2, CA3 and DG regions of PNV-exposed rats and control rats. Anti-NeuN upregulation reached significance at 24 h only for neonates as follows: 88% in CA3 (** *p* ≤ 0.01) > 65% in CA2 (* *p* ≤ 0.05) > 50% in DG (* *p* ≤ 0.05) > 46% in CA1 (* *p* ≤ 0.05). The NeuN level of envenomed adults was similar to their baseline values. Age-related differences demonstrated that neonate animals showed a tendency to exhibited higher levels of NeuN than adult rats which also reached significance at 24 h (^#^ *p* ≤ 0.05; ^##^ *p* ≤ 0.01; ^###^ *p* ≤ 0.001).

Western blot analyses of NeuN showed an 18% decrease at 5 h (** *p* ≤ 0.01) and a 15% increase at 24 h (** *p* ≤ 0.01) in PNV-treated neonate rats (Figure 5D). In adults, NeuN level was 20% downregulated at 2 h (** *p* ≤ 0.01), and was similar to baseline values at 5 h and 24 h. There were age-related differences between PNV-treated adult and neonate rats at 2 h and 5 h, with neonates showing higher levels than adults, as illustrated in Figure 5 (26%, ^##^ *p* ≤ 0.01 and 20%, ^#^ *p* ≤ 0.05, respectively).

## 3. Discussion

Despite clinical and experimental studies having shown that *P. nigriventer* spider envenomation induces neuroexcitotoxic signals, and that the venom disrupts the blood-brain barrier, causes reactive astrogliosis, neuroinflammation, and activates neurons [4,5,15,16], little is known about regulatory mechanisms triggered in the brain shortly after envenomation. 

VEGF type A, studied herein, is a hypoxia-inducible glycoprotein with a pivotal pro-angiogenesis regulator role [17,18]. VEGF is promptly upregulated in response to minimal changes in oxygen [19] and is therefore considered to be a neurotrophic/neuroprotective cytokine against brain damage [17,20,21]. VEGF translational regulation is accomplished through endogenous mechanisms that activate the oxygen-regulated α-subunit of hypoxia-inducible transcription factor-1 (HIF-1) [22]. In rats, intoxication by PNV includes neuroexcitability, respiratory distress, and convulsion [5,13,14], which all together lead to the loss of energy homeostasis in the CNS. Although both neonate and adult rats presented similar signs of intoxication, the results of the present study suggest that, following exposure to PNV, neonate rats seem to be less capable than adult rats in activating VEGF, Flt-1 and Flk-1 promoters, to produce mRNAs with efficient translational activity.

In fact, in neonates, only Flk-1 mRNA and Flt-1 protein increased, with this occurring later, at 24 h, while VEGF and Flt-1 mRNAs and VEGF and Flk-1 proteins remained unaltered. In contrast, in PNV-treated adult rats, VEGF mRNA and Flk-1 mRNA expression increased at 5 h, and Flt-1 mRNA increased at 5 and 24 h. The absence of changes and/or delayed response by envenomed neonates may suggest immature endogenous mechanism for activation of HIF-1 and translational regulation of VEGF, a hypothesis that requires further examination. Until now, studies of the effect of PNV on BBB have been conducted only with adult rats, but age-related differences have been recently reported for both rats [14,23] and humans [4].

The changes in the VEGF/Flt-1/Flk-1 system shown here were concurrent with hippocampal microvascular permeability, evidenced by perivascular edema and supported by expressional decreases of proteins associated with BBB, such as occludin, β-catenin and laminin. These findings are in line with previous quantitative results that have shown BBB disruption, vasogenic and cytotoxic edema in the hippocampus and cerebellum of rats given PNV [5,13,15,16]. Interestingly, redistribution of occludin, β-catenin and laminin occurred later in neonates (5 h) than in adults (2 h), and changes in their expression was in general less pronounced in neonates than in adults. Such findings substantiate the view that endogenous regulatory mechanisms against PNV toxicity seems to be more precarious in neonates than in adults. The data obtained in the present study agree with study showing that BBB in suckling rats, like P14, is immature and that differentiation of endothelial cells of brain vessels is not yet accomplished [24].

Another study suggests that transcription factors such as HIF-1 are an upstream mediator of junctional proteins during hypoxia, which may involve VEGF induction and expression [25]. Like HIF-1, VEGF at the BBB is considered a permeability mediator [11]. This is in keeping with VEGF function as a potent inducer of vascular permeability during strokes, ischemia and other conditions of oxygen brain deprivation. Additionally, changes in BBB-associated proteins have been reported in similar pathological states; and perivascular edema has been related to BBB leakage [8,26]. Activated Flt-1 and Flk-1 receptors stimulate a variety of signaling pathways and extensive biological responses in endothelial cells [27], but not exclusively. Studies have shown VEGF participation in vascular, glial and neural cells, the glio-neurovascular unit (g-NVu), to which BBB is integrated [28,29]. In previous studies, the authors of the present study have shown that PNV activates neurons (Fos induction) and causes neuroinflammation (induction of pro-inflammatory cytokines) [15,16], hence demonstrating that the venom affects all cell types of the g-NVu.

The reduction at 5 h of NeuN expression in the hippocampus of PNV-treated neonates followed by increase at 24 h (see WB- and immunochemistry-based data) could be associated with the upregulation of Flt-1 expression and Flk-1 mRNA at the same 24 h in these animals. The release of neurotrophic factors could result in a higher number of living neurons. VEGF signaling through Flt-1 and Flk-1 receptors has been positively implicated in the synaptic plasticity during adulthood, developmental and post-developmental stages of neurons; also current knowledge refers the role of VEGF in both the synapse formation and elimination implying a differential interaction of the neurotrophic factor with different subtypes of neurons [30]. Whether these differential effects correlate with the expression of specific sets of VEGF receptors in groups of neurons was not investigated yet. NeuN is a central element of the neuronal nuclear matrix that is only expressed in post-mitotic neurons [31]; it has been used as a marker of mature neurons and neuron viability [32].

Pyramidal neurons are the most abundant neuronal type located in forebrain structures, including the cerebral cortex, hippocampus and amygdala. Their location in the forebrain region indicates involvement with higher cognitive function. Conversely, the dentate gyrus of the hippocampus is a key relay station, common to all animals, which controls the transfer of information from the entorhinal cortex to the hippocampus proper [33,34]. Dentate gyrus granule neurons play a seminal role in this process, as they receive and integrate entorhinal synaptic inputs. The complex is involved in seizure-like events. Both pyramidal dendrites and granule dendrites possess *N*-methyl-d-aspartate (NMDA) receptors, and Na^+^ and Ca^2+^ channels, and therefore the possibility of being affected by ion channel acting-neuropeptides of PNV [3] is not excluded. Neuropeptides of PNV contain a calcium channel antagonist that blocks glutamate exocytosis but also inhibits glutamate uptake [35,36,37] in synaptosomes. VEGF modulates NMDA receptor activity in cerebellar granule cells through Src-family kinases before synapse formation [38] reduces calcium influx via inhibition of the Ca^2+^ channels in rat hippocampal neurons [39] and controls epileptic activity by influencing glutamatergic and gamma-aminobutyric acid (GABA)ergic neurotransmission [40,41]. Taking into account the fact that pyramidal and granule neurons NeuN(+) are VEGF(+), Flt-1(+) and Flk-1(+) cells, and considering the pharmacological characteristics of PNV, the changes in the expression of these proteins in the course of envenomation may represent their potential involvement in the hippocampus at the initial phases of envenomation by PNV. However, such supposition requires examination since the findings here found could be causal in nature.

Finally, the unexpected reactivity of anti-Flt-1 and Flk-1 in the nuclei of neurons is interesting. Like VEGF, the receptors typically exhibit a membrane-bound location. However, several studies have demonstrated that receptors can be translocated to the nucleus in both physiological and pathological conditions. Translocation of functional growth factor receptors to the cell nucleus has been considered a novel mechanism for growth factor regulation. Ligand binding can internalize the activated receptors to downregulate signaling through degradation of the ligand/receptor complex (ubiquitinylation) or via endocytosis of the receptor itself [42,43]. Although unclear, it is possible that the age-related differences in the expression of VEGF and its tyrosine kinase receptors in response to PNV observed herein may reflect differences in the internalization and subsequent nuclear trafficking of the receptors, thereby inpacting cellular processes in the hippocampal tissue.

## 4. Experimental Section

### 4.1. Animals and Venom

Male Wistar rats (*Rattus norvegicus*) aged three weeks were obtained from the Multidisciplinary Center for Biological Investigation at the State University of Campinas (CEMIB/Unicamp). They were housed under standard animal colony conditions, 5 per cage, at 23 °C on a 12 h light/dark cycle, with lights on at 6 a.m. and with free access to food and water until reaching 8–10 weeks old. At least 24 h before the experiment, the animals were transported in their home cages from the animal colony to the laboratory and allowed to habituate. Male Wistar rats on post-natal day 14 (P14) were taken directly from CEMIB to the laboratory, and experiments were done in the next day. Lyophilized *P. nigriventer* crude venom (PNV) was stored at −20 °C, until use. 

### 4.2. Envenoming Procedure

All experimental procedures were approved by the Institutional Committee for Ethics in Animal Use (CEUA/IB/Unicamp, protocol n. 2403-1) and the experiments were carried out according to the established by the Brazilian Society of Laboratory Animal Science (SBCAL) guidelines for animal use. P14 animals (*n* = 4–6) and 8–10 weeks old animals (*n* = 4–6) received a single intraperitoneal (i.p.) injection of PNV (1.7 mg/kg in 0.5 mL of 0.9% sterile saline), while the control group was given the same volume of vehicle [14]. Two, five and 24 h after saline or PNV i.p. administration, the animals were anesthetized and the hippocampus immediately removed. Neonate rats were used for comparison with adult rats, since severe accidents by *Phoneutria* generally occurs in children [4]. Time limits of 2, 5 and 24 h corresponded to periods of peak of intoxication, beginning of clinical recovery and no sign of intoxication at all, respectively [14].

### 4.3. Immunohistochemistry and Image Analysis

Anti-VEGF (1:50, mouse monoclonal, sc-7269), anti-Flt-1 (1:500, rabbit polyclonal, sc-316) and Flk-1 (1:50, rabbit polyclonal, sc-315), all from Santa Cruz Biotechnology (Santa Cruz, CA, USA), and anti-neuronal nuclear antigen (NeuN) (1:1000, rabbit polyclonal, ABN78, Millipore, Billerica, MA, USA) immunohistochemistry were performed in coronal sections of the hippocampus, as previously described [14]. Negative control was done with 1% PBS-bovine serum albumin (BSA) but without the primary antibody.

NeuN levels were determined as previously described [14], using the free access GIMP 2.6.4 software (GNU Image Manipulation Program, CNE, Free Software Foundation, Boston, MA, USA).

### 4.4. Western Blotting (WB)

After 2, 5 and 24 h of PNV or saline injection, the animals (*n* = 6/time), were anaesthetized by CO_2_ inhalation and killed by decapitation. The hippocampi from each group were quickly dissected and homogenized in an extraction buffer (10 mM EDTA, 2 mM PMSF, 100 mMNaF, 10 mM sodium pyrophosphate, 10 mM NaVO_4_, 0.1 mg of aprotinin/mL and 100 mMTris, pH 7.4). Cellular protein was quantified by Bradford assay (Bio-Rad, Hercules, CA, USA), then 40 µg of the cleared lysates were separated on 8% (β-catenin, Flt-1, Flk-1, laminin and occludin) or 12% (VEGF and NeuN) SDS-PAGE and electrotransferred onto nitrocellulose membrane (BioRad). Total cell lysates were prepared and analyzed by Western blotting, as previously described [7]. Antibodies were specific for rabbit polyclonal antibody against Flt-1 (1:500, sc-316), Flk-1 (1:250, sc-315, Santa Cruz, CA, USA),laminin (1:500, L9393, Sigma Aldrich, St. Louis, MO, USA), NeuN (1:2000, ABN78, Millipore, Billerica, MA, USA); mouse monoclonal antibody against β-Catenin (1:600, sc-7963) and VEGF (1:500, sc-7269) (Santa Cruz, CA, USA), β-actin (1:1,000, A2228, Sigma Aldrich) and goat monoclonal antibody against occludin (1:500, sc-8144, Santa Cruz, CA, USA). Bands were visualized using chemiluminescence reagent (Thermo Scientific, Waltham, MA, USA). For quantification, the density of pixels of each band was determined by the NIH Image J 1.45s software (available at ftp from zippy.nimh.nih.gov/ or from http://rsb.info.nih.gov/nih-image; developed by Wayne Rasband, NIH, Bethesda, MD, USA). The results of each protein were confirmed in three sets of experiments, and data were normalized using the respective loading controls. Values were normalized to the corresponding value for β-actin and expressed as a ratio.

### 4.5. RNA Isolation and Real-Time Quantitative Polymerase Chain Reaction (qPCR)

Total RNA was isolated from the hippocampus of each group using trizol reagent (Life Technologies, Gaithersburg, MD, USA). Primers used in this study and their respective assay identification numbers in the Applied Biosystem catalog were: VEGF: Rn01511601_m1, Flt-1: Rn00570815_m1, Flk-1: Rn00564986_m1. The levels of VEGF, Flt-1 and Flk-1 mRNA were quantitated relative to amplicon-specific standard curves by qPCR using 50 ng total RNA in triplicate and analyzed on an ABI Prism 7500 sequence detector, using a TaqMan^®^ Universal Master Mix. The optimal concentrations of cDNA and primers, as well as the maximum efficiency of amplification, were obtained by five-point, two-fold dilution curve analysis for each gene. Each PCR contained 3.0 ng of reverse-transcribed RNA, 200 nM of each specific primer, SYBR SAFE PCR master mix, and RNase-free water to a final volume of 20 µL. All samples were run in triplicate with water as a no-template control and GAPDH as an endogenous control. Real-time data were analyzed using the Sequence Detection System 1.7 (Applied Biosystems, Carlsbad, CA, USA).

### 4.6. Statistics

Student’s *t*-test was used for comparisons between PNV and control data. Additionally, three-way ANOVA test was used to determine the influence of three variables (treatment (control and PNV), age (P14 and 8–10 weeks) and time (2, 5, and 24 h)) on the resulting outcome. The data were expressed as the mean ± SEM; a *p* ≤ 0.05 indicated significance.

## 5. Conclusions

Overall, the data showed that the changes in the expression of VEGF, Flt-1, Flk-1 and their respective mRNAs in the hippocampus of rats administered with the venom of *P. nigriventer* spider were time- and age-dependent. These changes were concurrent with decreased proteins associated with BBB, occludin, β-catenin and laminin, simultaneously with the appearance of perivascular edema. Neonate rats seem to be more susceptible than adult rats. PNV-treated neonate rats showed increased NeuN expression. All the expressional changes showed age-related pace, with adult response more precocious (2 h) than that of neonates (5 h). Together, the findings suggest an interdependent and suggestively time-coordinated sequence of events. VEGF, Flt-1, Flk-1 and NeuN were expressed by pyramidal and granule neurons. Flt-1 and Flk-1 atypically exhibited nuclear translocation, with this hypothetically being a potential mechanism for regulating VEGF. Studies have shown that PNV induces excitotoxic signs which follow BBB breakdown and probably central energy unbalance. PNV possesses ion channel-acting neuropeptides that interfere with neurotransmission and glutamate handling. Future pharmacological studies are needed to reveal which interdependent signaling pathways underlie VEGF and the intracellular tyrosine kinase domains of Flt-1 and Flk-1 in the neurotoxic manifestations elicited by PNV. This will elucidate the actual role of VEGF in PNV-related hippocampal changes.
